# Nurses’ perspectives of taking care of patients with Coronavirus disease 2019: A phenomenological study

**DOI:** 10.1371/journal.pone.0257064

**Published:** 2021-09-03

**Authors:** Sarath Rathnayake, Damayanthi Dasanayake, Sujeewa Dilhani Maithreepala, Ramya Ekanayake, Pradeepa Lakmali Basnayake

**Affiliations:** 1 Department of Nursing, Faculty of Allied Health Sciences, University of Peradeniya, Peradeniya, Sri Lanka; 2 Department of Paediatrics, Faculty of Medicine, University of Peradeniya, Peradeniya, Sri Lanka; 3 School of Nursing, Kandy, Sri Lanka; University Hospital Of Ulm, GERMANY

## Abstract

The pandemic of Coronavirus disease 2019 (COVID-19) has brought significant pressure on nurses globally as they are the frontline of care. This study aimed to explore the experiences and challenges of nurses who worked with hospitalised patients with COVID-19. In this qualitative study, a purposive sample of 14 nurses participated in in-depth telephone interviews. Data were analysed using Colaizzi’s phenomenological method. Five key themes emerged: (1) physical and psychological distress of nurses, (2) willingness to work, (3) the essential role of support mechanisms, (4) educational and informational needs of nurses and (5) the role of modern technology in COVID-19 care. Although the provision of care led to physical and psychological distress among nurses, with their commitment and professional obligation, it is a new experience that leads to personal satisfaction. Guilty feeling related to inefficiency of care, witnessing the suffering of patients, discomfort associated with wearing personal protective equipment (PPE), work-related issues (e.g., long hour shifts), negative impact to the family and rejection by others are the leading distress factors. Religious beliefs, including keeping trust in good and bad merits, have become a strong coping mechanism. Addressing distress among nurses is essential. The reported learning needs of nurses included skills related to donning and doffing PPE, skills in performing nursing procedures and breaking bad news. Nurse managers need to pay special attention to expanding training opportunities as well as support mechanisms, for example, welfare, appreciations and counselling services for nurses. Modern technology, particularly robots and telecommunication, can perform an essential role in COVID-19 care. The establishment of timely policies and strategies to protect health workers during a national disaster like COVID-19 is needed.

## Introduction

Coronavirus disease 2019 (COVID-19) is a respiratory infectious disease caused by a newly identified coronavirus named SARS‐CoV‐2 [1, 2]. Health workers, especially nurses, have to play a significant role in combating this health problem on both preventive and curative sides. A recent systematic review identified that nurses have a pivotal role in healthcare when responding to infectious disease pandemics and epidemics [3]. Koh et al. [4] report that facing emerging respiratory diseases is an unavoidable health hazard for nurses who are in the frontline of care as nurses have to live, experience and accept this risk. Caring for patients with COVID-19 demands more knowledge and training [5]; however, the literature supports that nurses provide this care without adequate expertise [6]. Moreover, several studies have explored that nurses experience extra pressure, burden and psychological problems during global respiratory outbreaks (e.g. Severe Acute Respiratory Syndrome [SARS], H1N1 influenza, Human Swine Influenza and Middle East Respiratory Syndrome [MERS]) [7–13]. Therefore, nurses need continuous support and training to improve their preparedness and efficacy of crisis management as well as to cope with psychological problems and safeguard their well-being [3, 6].

However, there is limited evidence related to nurses’ experiences concerning caring for patients with COVID-19 globally. The available studies have mainly focused on exploring physical and psychological distress [5, 6, 14]. Liu et al. [6] have reported that health workers, including nurses dedicated to combating this pandemic while they experienced physical and emotional stress. There is a study that examined the overall perception of nurses towards COVID-19 care, and it identified challenges faced by nurses, for example, feeling of inefficiency, stress, fatigue, dilemma concerning care delivery and problems associated with using personal protective equipment (PPE) [15]. In a crisis like COVID-19, it is difficult to formulate a well-established evaluation plan; therefore, post hoc reflection of health workers helps to manage future crises effectively [9]. Therefore, further exploration of experiences, particularly the overall experiences of nurses who cared for patients with COVID-19, is essential. This study aimed to explore the experiences of nurses who cared for patients diagnosed with COVID-19 during the initial period of the crisis in Sri Lanka.

## Materials and methods

### Study design

This qualitative study employed Colaizzi’s phenomenological approach [16].

#### Participants and recruitment

Participants were nurses who took care of patients with COVID-19 in public hospitals for COVID-19 patients in Sri Lanka. A purposive sample of nurses was initially recruited through social media (i.e. Facebook). Then, the snowballing sampling method was applied to recruit potential participants. Data saturation was considered to determine the sample size [17]. Nurses who cared for at least one patient diagnosed with COVID-19 in public sector hospitals, who could speak in Sinhala, volunteers who were willing to participate in this study, and who were able to articulate their experiences were included. Nurses who worked in the private sector were excluded.

#### Data collection procedure

In-depth telephone interviews were conducted by the first researcher during June 2020, using an interview guide developed by the research team based on the literature and aim of this study (Table 1). The telephone method helped to collect data during the curfew period with travel restriction. After identifying the potential participants, we distributed written information sheets and consent forms via electronic media (i.e., email and WhatsApp). Possible time for both parties was set. Interviews were digitally recorded with prior permission from the participants. The duration of the interviews was ranged from 50 minutes to 75 minutes.

**Table 1:** Interview guide.

1	Could you tell me about your work experiences as a nurse?
	Probe: education, workplace, special training received
2	Could you tell me what kind of experience did you have before providing care for a patient with COVID 19?
	**Probe:** preparation (psychological, physical), formal training received, guidance and support received
3	Could you tell me what kind of experience did you have when providing direct care for a patient with COVID 19?
	**Probe:** preparation, performing procedures, communication with patients, supporting the patient’s physical and psychological needs, physical and psychological problems faced by nurse
4	Could you tell me what kind of experience did you have after providing care for a patient with COVID 19?
	**Probe:** physical, emotional and psychological responses, the responses received from peers, co-workers and family, moving to community/public
5	Could you tell me what type of support you received to provide successful care for a patient with COVID 19?
	**Probe:** support received, counselling
6	Could you tell me what barriers and challenges did you experience when providing care for a patient with COVID 19?
	**Probe:** barriers, challenges
7	Is there anything else you would like to add to today discussion?
8	Have you any question related to today discussion?

#### Ethical considerations

We obtained ethical approval for this study from the Ethical Review Committee, Faculty of Allied Health Sciences, University of Peradeniya, Sri Lanka. Verbal informed consent was sorted before data collection and recorded as a part of the telephone interviews.

#### Data analysis

Three members of the research team transcribed digital audio files. All personal identifiers were removed from the transcripts. Participants received the transcripts to check their accuracy and resonance with their experiences [18]. Based on Colaizzi’s phenomenological approach [16], relevant themes were identified. When writing the detailed description, relevant quotes were translated into English from the Sinhala language by two researchers and the consensus was achieved. In the reporting of this study, we followed the consolidated criteria for reporting qualitative research (COREQ) checklist (S1 Appendix) [19].

#### The trustworthiness of the study

The four-dimensional criteria were applied to ensure the trustworthiness of this study [20, 21]. To achieve *Credibility*, participants received a copy of the transcription for member checking. Persistent observation of data was ensured by identifying themes and sub-themes, including reading and re-reading data and identifying statements and meanings relevant to nurses’ experiences. Additionally, participants received a summary report based on findings to check whether the analysis captures their experiences. For example, P13 said that results are a representation of the experiences and challenges they experienced during providing COVID-19 care. *Transferability was* assured by providing adequate contextual information (setting, sample, sample size, sample strategy, socio-demographics, inclusion and exclusion criteria and interview procedure). *Dependability* was achieved by using accepted standards i.e., Colaizzi phenomenological approach [16] in data analysis. To assure *conformability*, the first researcher identified the potential statements and meanings for the first transcription, and these meanings were discussed with other researchers for consensus. A continuous discussion was endured for new meanings. The first and second researchers categorised meanings and identified themes and sub-themes. A consensus was achieved for the final themes among team members.

## Results

### Demographic characteristics

Table 2 shows the demographic characteristics of the participants. The sample consisted of one male and 13 female nurses.

**Table 2:** Demographic information of participants (n = 14).

Characteristics of nurses	Number (%)	Mean ± SD
**Age**		33.36 ± 6.3
**Gender**		
Male	1 (7.1)	
Female	13 (92.9)	
**Marital status**		
Married	10 (71.4)	
Single	4 (28.6)	
Widowed/divorced	0	
**Highest education in nursing**		
Diploma in Nursing	10 (71.4)	
BSc in Nursing	4 (28.6)	
**Years of experience in the nursing career**		8.14 ± 6.11
**Type of hospital**		
Newly established for COVID-19 care	10 (71.4)	
Not newly established	4 (28.6)	

### Findings

Five key themes and their attended sub-themes were identified (Table 3). Nurses were de-identified in reporting (e.g., P1).

**Table 3:** Themes and sub-themes.

No	Themes	Sub-themes
**1**	Physical and psychological distress of nurses.	Fear towards COVID-19
		The negative impact on family
		Social stigma and discrimination
		Witnessing patients’ experiences
		Guilty feeling related to inefficiency of care
		Work-related physical and psychological discomfort
		Coping mechanisms
**2**	Willingness to work	Sense of professional obligation
		Provision of care as a new experience
		Personal satisfaction among nurses
**3**	Educational and information needs of nurses	Need for prior training and education
		Learning needs of nurses
		Learning strategies
**4**	Essential role in support mechanisms	Need for adequate resources
		Need for welfare
		Lack of appreciation and incentives
		Need for professional counselling
		Support network of nurses
		Role of nurse managers and administrators
		Need for timely policies
**5**	Modern technology in COVID-19 care	Robots in direct patient care
		Telecommunication in patient care
		Modern technology in information seeking

### Theme 1: Physical and psychological distress among nurses

This theme discusses the physical and psychological distress experienced by nurses.

#### Fear towards COVID-19

Participants identified COVID-19 as a frightening disease. They stated that they were at increased risk of getting the infection; this risk was unavoidable, which led to increased fear of exposure to the virus. “Really, it is a risk. No matter how many safety precautions we take. If there is a slight mistake, we need to be afraid.” (P13). One nurse said that she heard several deaths due to COVID-19 among health workers around the world, and it worsened her feeling of scared. Some participants said that they were panicked when they heard the first diagnosed patient was coming to the unit and showed extreme fear when admitting the first patient. One participant said that she had a hallucination like feeling after the provision of care. “… Sometimes it’s like hallucination while on duty in the ward…sore throat. When I go home, I just feel like that… hurts a lot” (P2). Participants stated that they followed precautionary measures to maintain their health and to prevent from COVID-19. The reported measures were regular hand washing, regular temperature checking, drinking hot water, using traditional remedies like coriander and ginger mixed water, taking cod-liver oil, taking a high dose of Vitamin C, taking anti-histamine, having hot water baths and steam inhalation.

#### The negative impact on the family

The majority of participants showed a feeling of fear related to being a potential carrier for family members. “I was a little scared that it would happen to me, but I scared, I would give this terrible disease to my husband, then to his family, the mother is too old…” (P6). They said that their family members were too scared, and dealing with those suffering was intolerable. Returning home and living with family members in the same house, living in hospital accommodations and looking after kids were some other issues reported. Participants reported that separation from family members, especially from their kids, was intolerable. “At that time, I came home, and my two babies were next door with my sister. I’m over here. I stand in the yard looking at my two children. How they are in that yard. It’s really hard to remember” (P14).

#### Social stigma and discriminations

Participants said that they were rejected by peers, co-workers, family members, neighbours and society, making them frustrated. One participant said that when she worked in the COVID-19 unit, others looked at her like a patient with COVID-19 positive. “At that time, others looked at me; it was just like corona to me…” (P14). Another participant said that not only the general public, health staff also rejected them. “..Oh. Really sad. So when the health staff does the same. You don’t need to talk about ordinary people” (P13). Moreover, few participants said that they and their families were rejected by the neighbours, shops, and taxi drivers. “After this hospital was named as a corona hospital, there were rejections from the shops around” (P11). The majority stated that they did not tell others that they were working in COVID-19 units, and this condition led to limited social interactions and self-isolation. Some participants noted that rejection might be attributed to social stigma and the frightening nature of COVID-19.

#### Work-related physical and psychological discomfort

Work-related factors, including lack of staff, working long hour shifts, increased workload, and inadequate rest time, were other main factors that led to physical tiredness and psychological burden. “In some shifts, the workload is too much for a single nurse; then, there was no time, we didn’t even have water or cup of tea” (P13). “There were only two nurses for 15 patients in this ward, but we had to work for about 50 patients. It was very difficult for us and tired. When we finished our duty, we were exhausted” (P2). Therefore, they highlighted that they require adequate rest time to improve their immunity.

Additionally, wearing PPE is one of the main factors that led to the physical and psychological burden. Reported physiological discomfort included difficulty breathing, excessive sweating, headache, back pain, skin damage and pressure on the nasal bridge due to strips of goggles, vomiting, fainting and visual disturbances. “…that goggle.., put on a cap.., it’s too much to bear, the day before I had a headache for a day and a half or two… and back pain, we walked in boots …, It’s hard.., there is a big discomfort in the body…” (P3). Participants further reported mist due to facemask and googles made difficulties in cannulation and drawing blood. In terms of psychological burden, one participant stated that wearing a PPE first time was a frightening experience. “I was so scared. I mean, it was hard to breathe when I put on an N95 mask. I felt very restless for a while. Then, I thought for a while, and that feeling went away a bit. Then, I went to collect blood…” (P12). They further said that they had an uncertainty related to protection received from PPE. They said that they had to take additional measures before donning PPE, for example, eating and drinking adequate water and going to washrooms, adding emotional discomfort. Moreover, all participants identified removing PPE as a very relaxing experience. One participant said that it might be due to physical relaxation or releasing from the risk of contact with patients. Another participant stated this experience as ‘like attaining Nirvana’, (i.e., a Sinhalese saying related to a feeling of sheer relaxation). “…after removing the PPE…, like going to Nirvana. Um… .it’s like going to Nirvana…” (P2).

#### Witnessing patients experiences

Participants said that they witnessed the suffering of patients, including their fear of death. “…the fear in the heart of the patient when he was admitted. This is a fatal disease. All those patients told the story; we will die or what will happen to us…” (P12). They further stated that patients were very anxious, and some patients reported depressive symptoms, especially when they received positive PCR results. “…those guys… . yes, psychologically really upset. Let’s assume that when a patient is discharged, from the same set, if one patient cannot go home, that means he is the only positive, they mourned… like in a funeral…” (P8). They further said that older people and people with chronic diseases were terrified, and older people were helpless in the wards as nurses could not reach them all the time. Several participants stated that informing negative results to patients was the happiest experience for the patients and nurses. “He is 27 years old boy…I said, brother, your report is negative, now you can go, I really said ‘brother’… that boy was so worried. He cried when I told him that his PCR was negative, that’s happiness…” (P14). “I told the patient that your report is negative and you can go home in the evening. This is the best, happiest last moments out there…” (P2).

#### Guilty feeling related to inefficiency of care

Although they were empathetic towards patients, participants stated that they had to limit care due to strict guidelines imposed. This situation caused a reduction in direct care time that led to the inefficiency of care. For instance, one participant stated, “... I did only whatever works need to do. We can’t go inside always without PPE…” (P11). Many participants said that wearing PPE limited the establishment of a good nurse-patient relationship. “…poor people. I meant they don’t see us. Really, they don’t know who we are. They know we’re nurses because we say we’re nurses.” (P2). They further stated that the provision of care with these limitations led to a guilty feeling as they were unable to provide adequate care compared to usual care. “Those people are terrified. We are less likely to get too close to a patient, even to reassure, when that happened, I felt sad when I couldn’t do that” (P11).

#### Coping mechanisms

Many nurses highlighted that they believed their religion and followed religious activities before and after their duty shifts. “I am a Buddhist, of any religion, everyone is bound to give nursing care by their religion. I think my religion has taught me a lot about this. If we do something good, our parents and we will get something good. I really believe this” (P11). Some nurses believed that everything happened according to ‘good’ and ‘bad’ merits, and if they did a fair job, they would re-paid it as a prevention from COVID-19. “No matter what we do, you get it as good or bad merit whether you are a Catholic, a Muslim, a Buddhist or whatever. Although the way of saying ‘good and bad merit’ in each religion has changed, the way of receiving is the same”(P2). As reported by nurses, the main group who shared their suffering were nursing peers and friends. “We all talk together… we can’t go home. Sometimes in the quarters, talking to two or three people, (laughing)… and sharing our grief” (P3). Other reported methods were crying, trying to hiding uncomfortable thoughts (i.e., repression) and rationalisation. “…at the same time… No, it will not happen. He was also wearing a mask; I was also wearing a mask. This did not happen…” (P1).

### Theme 2: Willingness to work

This theme highlights nurses’ willingness to provide care, including their sense of professional obligation.

#### Sense of professional obligation

Nurses highlighted a sense of obligation for work. Some participants reported that they should provide this care because they were nurses. “It is not right to leave this time due to personal matters… . I am a nurse. I get paid” (P1). They showed their dedication and professional commitment to providing care. One participant said that her fellow nurse ran to a patient without completing PPE donning when the patient self-remove her endotracheal tube. “The patient self-removed the tube. The saturation of the monitor is dropping. Monitors are alarming. Now, she was half-dressed…, this nurse suddenly ran without goggles and boots” (P 14). Participants stated that this was an opportunity to serve the community and mother country that everyone cannot do. “I think I was able to do something better than others. We were able to do a lot for patients’ happiness… (P9). “…I had a feeling that I also participated in that national mission…” (P6).

#### Provision of care is a new experience

As COVID-19 care is a newly emerged area, some participants stated about their motivation to provide care. They were curious and had a desire to work with patients. “Actually, I had to see how she (patient) was…, how we could deal with her…?” (P3). “It was a different experience … that’s why… at that time, it felt like to do something… that means I felt to go to COVID care unit” (P 7). Participants further viewed that it was a new experience for their professional life and an opportunity to learn and test new care strategies and protocols. “Everything is a new procedure and a new experience” (P12). “As a staff member, I tried my best to reduce patients’ stress. It feels like weird nursing care, not normal nursing. Did it in a new way. We had to adapt to the problems that arose” (P11). One participant noted that it was the best experience she had in her nursing life.

#### Personal satisfaction of nurses

Although the provision of care was a terrible experience for them, many participants stated that they were delighted with the given care at the end of their placements. They used the words “happy”, “pleasure”, or “proud” to explain their satisfaction. “It’s nice to be involved with something like this. It’s a pleasure. Everyone is scared. We, as nurses, are directly involved. Proud of it. Glad to be able to do something as a nurse that not everyone can do…” (P6). One participant said that she felt like a ‘hero’ when she first cared for a patient. Moreover, they stated that they were happy as mass media promoted and highlighted their contribution to the COVID-19 crisis.

### Theme 3: Educational and information needs of nurses

This theme encapsulates the educational and informational needs of nurses who cared for patients with COVID-19.

#### Need for prior training and education

All participants highlighted the need for prior training and education to provide care for patients with COVID-19. Few participants said that previous experiences related to providing care for H1N1 influenza and MERS were beneficial to provide successful care. “…When H1N1 was there, I was on duty in that ward. With that experience, I was not scared. I have even taken blood samples from H1N1 positive patients” (P2). Although some nurses had participated in related training programmes, some reported on inadequate or no prior training opportunities, and it increased their fear of patients and care. “We placed there; we had to do everything. But I didn’t have any training. I always felt I would contaminate because I didn’t know how to do” (P14). “It was a little training. To wear PPE and how to do the cleaning. But, when I went to the ward, I thought, it was not enough” (P10). One nurse stated that only demonstration was not adequate for wearing PPE, and nurses should have even one opportunity to wear a PPE kit before the actual practice. They highlighted that the basic nursing curriculum needs to address these aspects, and a sufficient number of nurses should be trained to confront future challenges in a crisis like COVID-19.

#### Learning needs of nurses

How to maintain safety during COVID-19 care, including donning and doffing of PPE, and performing nursing procedures, including collecting samples for PCR and disinfection, were the main care-related learning needs. “I did not even know how to wear the kit. N95 masks were not even in the ward (i.e. usual workplace). So, I was embarrassed because I did not know how to do” (P14). The other prioritised learning need was breaking bad news. Participants highlighted the challenges faced during informing positive PCR results to patients. “If we did fourteen PCRs, we say how many negatives and how many positives to patients. Only a few of these people were negative. Others were positive. We had people from different countries as well as from the Navy. It may vary from country to country” (P11). One participant said that they informed results to all patients as a group, while another stated that some patients wanted total confidentiality on positive PCR reports.

#### Learning strategies of nurses

Participants further explored various learning strategies that they used during this period. Some nurses have participated in the planned training programmes conducted at the National Institute of Infectious Disease (NIID), Angoda, and they said that it was beneficial in providing care. Some hospitals have arranged institutional-based in-service programmes before opening new units. The majority noted that peers, especially experienced and trained nurses, helped them to learn wearing PPE and other care. “There were nurses who were trained. I asked them and did the same” (P7). According to participants, self-directed learning was a robust method, and the internet and the media helped improve their knowledge of COVID-19 care. Additionally, participants highly valued the availability of care guidelines and protocols and receiving timely information enabled them to get new knowledge and provide safe care.

### Theme 4: Essential role in support mechanisms

This theme highlights the importance of support mechanisms, including workplace support and personal support networks for nurses.

### Need for adequate resources

Participants emphasised that a comfortable work environment was paramount in providing adequate care, and they highly valued the availability of sufficient human resources, other facilities and equipment. They highlighted that a pre-plan is essential when establishing units for people affected with COVID-19. One participant stressed that they had a sufficient number of supportive staff, and it was very helpful. “While there were positive patients, there was a larger group of minor staff than us. They gave good support in our works” (P13). Participants said that the availability of facilities, particularly adequate protective measures, cleaning facilities and bathing facilities, are essential to ensure their safety and occupational preparedness. “…to get the maximum care from us, we need to have adequate protection. If we do not receive those things, we are also afraid, and it is difficult to give good care” (P11). However, some nurses reported inadequacy of facilities and equipment; for example, inadequate PPE, poorly arranged patient and staff areas, lack of restrooms, lack of facilities for cleaning and bath, and inadequate facilities to communicate with patients.

#### Need for welfare during frightening health crises

The majority of the participants highlighted the need for welfare facilities, especially foods, transport and accommodation during a crisis. “We could have provided more facilities, food, transport, accommodation. We returned home daily. It’s hard. No problem, because none of us was COVID positive. If we came home while we were COVID positive…what will happen?” (P12). As reported by nurses, imposing curfew, travel restriction, lack of public transport, and limited shopping opportunities have limited their day-to-day life. Many emphasised that closing schools have created an additional burden to them as they had to look after their children while dedicating themselves to provide continuous care during this crisis. Some participants reported that managers of some hospitals paid attention to nurses’ welfare but expanding was essential.

#### Need for appreciation and incentives

Nurses highlighted a lack of appreciation for their tireless efforts in caring for patients from managers and administrators. However, they identified patients as the main appreciators. One nurse said that patients’ feedback and appreciations were stuck on the door and window glasses as poems. “When patients leave, they stuck poems on the glasses. They have written, they were very scared when they came. But they were treated well. Felt like they were at home, and they had everything they needed” (P10). Although few hospital directors and nurse managers appreciated them, the majority highlighted a lack of appreciations from authorities. “They said, they will send a letter, but, still I did not receive” (P12). The lack of incentives (e.g. additional allowances and risk allowances) was another important point highlighted by nurses. Moreover, some participants said they had economic issues and did not receive payment for their overtime duties. “We were labelled as health heroes. We know we worked. Although labelled, we did not receive our overtime and public holiday payment even” (P2). They said that this situation demotivated them.

#### Need for professional counselling services

Although some nurse managers supported to relieve the psychological stresses of nurses, many participants stated a lack of professional counselling services for both patients and staff who were in need during this situation. One participant said that she was depressed due to changing her workplace and placing her in a COVID-19 unit, but she did not receive any support to cope with her psychological concerns. Another nurse said that the establishment of counselling needs to be initiated at the very beginning. “I think it would be nice to have counselling for nurses at the very beginning. We had nurses who did not come for the duty” (P8).

#### The support network of nurses

Participants identified the support received from peers, co-workers, family, friends and neighbours as the main branches of their support network. Many junior nurses said that their senior nurses helped them in decision making. They appreciated the help received from the care assistants and junior staff who worked in the same units. However, participants said that staff who worked outside the COVID-19 care units had expressed extreme fear of COVID-19, and it has led to difficulties in getting their service done. The majority of nurses highly valued the support received from the family in terms of psychological and other supports. Some nurses said that family members encouraged them to go to work. “My husband always said you have gone to do good to people where many people are reluctant to go. You go. You do not go to do any wrong. Do not be afraid” (P14). Many participants said that family members and relatives looked after their kids during this challenging period. However, one participant reported a negative experience. “I live in my husband’s house. My husband’s mother left the house when I said I am working in COVID hospital” (P7). Some participants said that their friends and neighbours helped them bring food and other stuff to their houses, manage household works, and provide transport.

#### Role of nurse managers and administrators

Participants highlighted that hospital administration and nurse managers have a central role in COVID-19 care, particularly ensuring support for nurses. Nurses highly valued nurse managers’ positive leadership roles. Some participants identified nurse managers as experts because they used their prior experiences related to crisis management in organising COVID-19 care. The activities led by nurse managers, for example, doing basic risk assessment among nurses before placing to COVID-19 units, the arrangement of training programmes, granting leaves, arranging progress meetings, and arranging welfare, were highly appreciated. “Really, they are good. They helped us. They looked after patients as well as us. They asked what the problems are and what the shortcomings are. They arranged meetings to talk” (P9). However, some participants highlighted poor support received from and weak leadership qualities of nurse managers. They expected strong leadership qualities from nurse managers. One nurse said that when more patients come, nurse managers made decisions that benefited the institution than the staff. “Some of their answers, sometimes, to the advantage of the institution. Yes. Sometimes, they do not think about us at all. If not, they give the timely answers” (P2). Other main problems reported related to nurse managers’ role were placing nurses to COVID-19 units without asking their willingness or informing them, selecting only junior nurses, changing duty roster without informing, placing both husband and wife to COVID-19 care units, inadequate supervision, understaffing, and poor communication and coordination. One participant said that training was given to other nurses, but she had to work in the COVID-19 care unit without training. “At that time, I was really angry with the management. Others took the training, but we had to work” (P14). Many participants reported that they did not receive an opportunity to check PCR or did not receive a quarantine period, and they directly placed to the previous workplaces just after completion of duty at COVID-19 units.

#### Need for timely policies

Participants further highlighted the need for timely policies in managing the COVID-19 crisis. These policy concerns included government, institutional and unit-level policies, including pre-planning before opening hospital and units, recruitment and duty delegation, resource allocation, policies related to infection control, testing PCR and providing facilities and welfare. One participant said that there was a plan for quarantine for nurses where necessary. Another one said that there was an excellent discharge policy in the hospital. “We did not discharge a patient until three consecutive PCRs are ‘negative’. Only after three were negative, the patient was discharged” (P8). However, many participants reported that there was no good plan to place the nurses in COVID-19 units, and one participant said that immediate calling is not a successful method.

### Theme 5: Role of modern technology in COVID-19 care

This theme highlights the uses of modern technology in COVID-19 care.

#### Robots in direct patient care

Participants stated that they used robots to deliver foods, medicines and other stuff to patients. Participants said that using robots in direct patient care reduced contact time with patients, which was a big help for nurses. “That means we do not always collide with the patients. We had a robot. He is the one who sends all the foods to the patient. We only collide directly with the patient when we take blood. That’s why we have to go very rarely” (P7). “We did not have much contact. We went to the patient when we wanted to collect blood and PCR. All the foods and medicines were sent to patients through the robot. So, having a robot was a big help” (P12). However, one participant said that there was a guilty feeling when introducing robots to COVID-19 care as it limited the nurse-patients interactions. To escape this guilty feeling, this nurse further noted that she maintained communication at an optimal level when she went to collect blood from the patient. “There, we just had an upset. That means we can’t go to the patient. Then, when we went to collect blood, we definitely talked to the patient. That means we were talking to the patient rather than over the phone or robot at that time. The relationship was well maintained” (P12).

#### Telecommunication in patient care

Participants stated that modern communication methods, such as mobile phones, intercom system, and video camera systems, were used to improve nurse-patient communication, which helped to minimise exposure time to patients. “Most of the time, the patient and we communicated over the phone. So, we were not exposed. Besides, there was a video cam. We can talk to the patient even when robot went to the patient” (P2). One nurse highlighted the need for modern technology to improve communication with patients. “If there is a way to keep communication, a camera system, there is a way to talk about their problems and know their needs” (P4). One nurse stated that video records that included health education used to educate patients about COVID-19 and prevention. Additionally, nurses said that smart technologies, mainly smartphones, were promoted to patients as a way of communicating with their loved ones via video call.

#### Modern technology in information seeking

Participants stated that the internet was one of the main sources of information seeking. They said that websites and YouTube were used to acquire not only knowledge but also in learning the necessary skills required. One nurse who did not receive prior training stated that she used videos available on YouTube to know how to provide care for patients with COVID-19. “With the support of others, I found out by watching the videos on YouTube.” (P7). NIID has developed a video related to COVID-19 care, and many nurses have used it to learn the required skills. “I watched the video by IDH (i.e., a short name for NIID). How this should be treated. How to wear PPE…How to remove PPE” (P1). Additionally, social media has been used to share and receive information. One nurse said that when she was appointed to the newly established care unit, she contacted a nurse who worked in NIID through Facebook and got the necessary information and knowledge related to caring for patients with COVID-19. “A nurse at IDH, which means, I’m not her friend. I made her my friend on my Facebook. (Laughing). I asked details from her, how is the patient cared for?” (P2).

## Discussion

This study explored the experiences of nurses who cared for patients with COVID-419 in Sri Lanka, especially in the initial period of the crisis. To the authors’ knowledge, this study is one of the first studies to examine the overall experiences of nurses as the recent studies related to caring for patients with COVID-19 have focused mainly on physical and psychological distress [5, 6, 14, 22].

In line with recent studies related to COVID-19 care, this study reported a higher level of physical and psychological distress among nurses [5, 6, 14, 15, 22]. Fear towards COVID-19 has become one of the top reasons for their psychological distress. Recent studies report an extreme fear of COVID-19 among the general public [23] and health workers [6, 15]. Consistent with our findings, Kim [10] reported fear among nurses towards contagious respiratory diseases is inevitable. The highly infectious nature, the high morbidity and mortality rates, the nature of the novelty of disease [24], and the non-availability of drug or vaccine [14] have increased this fear. Similar to our study, fear of contact and transmission of the disease is a significant issue confronted by nurses during respiratory disease outbreak [6, 8]. In our study, nurses have paid special attention to maintain their health, and they have followed different measures to prevent the infection. This result is consistent with a recent study [14]. Extreme fear towards disease affects not only the psychological health of nurses but also patient care. To minimise the fear of COVID-19 among nurses, expanding education and training opportunities to improve knowledge and skills related to COVID-19 care is essential. Managers need to ensure a safe work environment for nurses in COVID-19 care units.

Similar to the findings of a recent study [15], this study reports fear of disease among nurses has been aggravated by being a carrier for the family members. Due to this situation, family members have shown extreme fear as they work in COVID-19 care units. Authorities need to ensure adequate precautions for nurses when they return to families. Additionally, the most reported causes for psychological stresses were separation from family members for an extended period and the inability to physically present to the family during this difficult time. Similar findings can be found in other studies [5, 22]. Improving resiliency among nurses is essential. Support networks for families of health workers who are in the frontline of COVID-19 care need to be expanded.

Consistent with the findings of a previous study [25], facing social stigma and discrimination, mainly rejection by others related to COVID-19 care found in this study, are common issues for nurses worldwide [26]. Stigma has led to label nurses as ‘disease carriers’ [24] and limited social interaction and isolation among nurses. The stigma associated with COVID-19 is a predictor of compassion satisfaction, burnout, and compassion fatigue among health workers [25]. The present study reported not only the behaviour of the general public but also the behaviour of staff who did not work in the COVID-19 frontline contributed to stigmatisation and discrimination. To minimise the possible stigma and discrimination, improving public awareness needs to be expanded.

Moreover, this study reports sadness, worries and feeling of guilt related to care provision among nurses. Sadness and worries are mainly attributed to witnessing patients’ suffering, and recent studies on COVID-19 care also reported a similar phenomenon [6, 15, 22]). COVID-19 signifies with rapid progression of symptoms with high mortality and often leads to death [27]. Evidence indicates that witnessing patients’ sufferings, especially the painful end of a patient, is one of the main sources of psychological pressure among nurses [22]. Moreover, this situation has increased by the guilty feeling of nurses because they have to provide limited care compared to usual care due to strict care guidelines imposed; for example, wearing PPE before contact with patients. Similar to the present study, the feeling of the inefficiency of care has been explored in a recent study [15]. These negative consequences are associated with burnout, compassion fatigue and reduced well-being of nurses [28]. Improving psychological resilience is essential to cope with these issues [24, 29], and psychological counselling for nurses are recommended [15].

Consistent with recent studies, this study further reported that understaffing, long working hours, shift work, and increased workload were associated with physical and psychological distress among nurses [5, 6]. World Health Organisation (WHO) [30] highlights that work-related factors are positively related to the development of fatigue, staff burnout, increased psychological distress and reduced mental health among nurses who cared for patients with COVID-19. Paying attention to improving work conditions for nurses is essential. Moreover, discomfort related to PPE was found to be one of the significant sources of physical and psychological stress of nurses in this study, coinciding with the recent findings concerning COVID-19 care [6, 14]. Difficulty in breathing, excessive sweating, headache, back pain, pressure on the nasal bridge due to strips of goggles, feeling of vomiting and fainting, and visual disturbances were the main problems identified. This study further identified that discomfort is increasing due to prolong usage of PPE, and a recent scoping review reported that prolonged use of PPE led to severe physiological discomfort; for example, skin breakdown among nurses [31]. To minimise the negative consequences of wearing PPE, nurse managers need to establish strategies for ensuring the safety of staff members by minimising the time required to wear PPE.

This study further explored the coping mechanisms used by nurses during this period. Interestingly, religious beliefs and practices and keeping trust in good and bad merits have become a powerful coping strategy among nurses. These beliefs help people to manage their stresses effectively compared to those who do not have religious practices [32]. Other reported coping strategies were sharing with peers, crying, repression and rationalisation. Among nurses on the front lines of COVID-19, strategies for coping with stress must be strengthened.

Although COVID-19 is a very frightening disease that led to physiological and psychological burden among nurses, similar to previous findings, this study reports the professional obligation, motivation and dedication of nurses who provide care for patients with COVID-19 [5, 15]. The commitment of nurses has been reported in previous respiratory outbreaks, for example, SARS [9] and swine flu [11]. Sun et al. [5] highlight that negative emotions are dominant in the early stage of the crisis, but positive emotions appear gradually in the later stage. Similar to this phenomenon, this study says that nurses view COVID-19 care as a new experience and leads to personal satisfaction at the end of their duty placements. Provision of care for patients with COVID-19 is a great opportunity for the professional growth of nurses that include honour and respect [5]. The present study reports that mass media portrays nursing positively, and nurses’ contribution has been highly valued globally. Consequently, authorities need to appreciate and promote nurses’ invaluable contribution; then, nurses can continue this care efficiently.

Similar to recent studies [5, 14], this study reports the importance of prior education and training for nurses during pandemics. Lack of knowledge is one of the main reasons for insecurity, and providing education on prevention and control of COVID-19 can reduce the psychological burden and insecurity among nurses [14]. This study reports inadequate or no prior training opportunities for nurses, and this situation leads to increasing fear and psychological distress related to providing COVID-19 care. Donning and doffing PPE, performing nursing procedures and breaking bad news were identified as prioritised educational needs of nurses. Especially, breaking bad news has been identified as a core component of communication concerning COVID-19 care, and demand for resources and support for effective bad news conversations is highlighted [33]. Education and training opportunities are needed to be expanded for nurses concerning the above learning needs in the Sri Lankan Context. This study further highlights the need for adding this content into basic curricula; therefore, nurse educators need to modify basic nursing curricula in Sri Lanka.

In addition to the availability of formal training programmes, peer learning, availability of care guidelines and protocols, self-directed learning, including the use of the internet and videos, such as a video developed by NIID, are the main learning strategies used by nurses. Promoting peer learning and ensuring timely care guidelines and protocols are essential. Self-directive knowledge has a promising role in health profession education [34], especially in emerging pandemic like COVID-19. Ensuring the availability of self-directive learning materials and providing facilities, for example, the internet and computers can promote nurses’ motivation for learning during health crises.

This study further highlights the support mechanisms available for nurses in the workplace and their personal life. Available support mechanisms of the workplace include the availability of adequate resources that ensure a safe work environment, welfare facilities, appreciations and incentives, and counselling facilities. WHO [30] also highlights the need for a healthy, safe and decent working environment for all health workers who provide care during the COVID-19 pandemic. Similar to previous studies, this study highlights the need for adequate resources, such as human resources, physical facilities, equipment, and PPE, to ensure a comfortable work environment [5]. This study further highlighted the role of the administrators and the need for timely policies in addressing the above aspects. Nurse leaders have an essential role in ensuring the safety of nurses [35]; therefore, the strong leadership qualities of nurse managers are crucial. Clinical leaders can be introduced to current practice settings in Sri Lanka.

In line with previous studies [5, 6, 14], this study identified the importance of support networks, including support from managers, peers, co-workers, family, friends and neighbours. The absence of sufficient support during infectious pandemics brings short-term and long term impact on nurses’ mental health [36]. Hence, authorities and media need to pay special attention to highlight the need for support for health workers during this crisis and strengthening the available support networks. Additionally, weaknesses in support strategies, especially welfare facilities for nurses, including accommodations, transport and meal facilities, were reported. Society has been limited due to curfew and lockdown, travel restrictions, closing shopping and limited transport. Therefore, expanding welfare for nurses should be prioritised. Moreover, our study reported a lack of appreciations, including incentives for nurses. In comprehensive workforce planning and development, appreciations and incentives are crucial to attract, retain and motivate health workers [37]. Establishing policies and strategies is essential to appreciate nurses’ hard work in the Sri Lankan context.

This study further explores the use of modern technology in COVID-19 care. One of the uses is telepresence robots. To the authors’ knowledge, this is the first experience of using robots to provide direct nursing care by nurses in the Sri Lankan context. Robotic systems can significantly reduce the transmission risks of infectious diseases for frontline workers because robots can provide care from a safe distance [38]. Nurses reported that robots were primarily used in serving foods and medicine. But, robots can be effectively used in other areas, for example, disinfection, measuring vital signs and assisting border controls in COVID-19 care [39]. Although nurses viewed that the use of robots in care helped to reduce direct contact time with patients, it led to a guilty feeling. With possible advantages, changing nurses’ perception towards robotic interventions in delivering care for frightening diseases like COVID-19 may be beneficial. More studies are recommended to examine user acceptance towards telepresence robots in direct nursing care in low-income countries like Sri Lanka.

Additionally, modern ICT, including internet, mobile technology, telephone, video technology with conferencing, internet-based education and videos, and social media, has been used in direct nursing care, informal education and information seeking. This study highlights the importance of those technologies in maintaining patient care while minimising possible contact. The literature emphasises the importance of modern technology in patient communication, workplace-based learning and increasing public awareness concerning COVID-19 care [40]. Therefore, nurses must be provided with the necessary support and facilities to use virtual technologies effectively. Skills training, including assessing patient’s non-verbal cues, emotional states and their understandings through virtual technologies, is essential [40].

### Limitations

We conducted telephone interviews. The absence of visual cues, the potential for the distraction of interviewees by environmental disturbances and technological issues related to telephones are some limitations in telephone interviews [41].

## Conclusion

This phenomenological study provides an insight into the experiences of a sample of nurses who took care of patients with COVID-19 in Sri Lanka. Although COVID-19 is a frightening disease with many negative impacts on nurses and their families, with their commitment and professional obligation, taking care of patients with COVID-19 is a new experience that leads to personal satisfaction among nurses. Physical and psychological distress among nurses is a common phenomenon due to worries related to witnessing the suffering of patients, guilty feeling related to limitations of care, work-related factors, discomfort associated with wearing PPE, negative impact to family and stigma and discrimination. Addressing psychological distress among nurses is a priority need. Hospital administrators and nurse managers have a significant role in making a comfortable work environment for nurses, including creating timely policies, providing adequate resources, training opportunities, comfortable shift methods, welfare, appreciation methods and incentives for nurses. Main support networks include the support received from management, peers, co-workers, family, friends, and neighbours. Strengthening these support mechanisms is essential. Previous education and training, as well as proper guidelines, are necessary to provide adequate care for patients with COVID-19. The main learning needs of nurses include donning and doffing of PPE, breaking bad news and performing nursing procedures. Expanding learning opportunities and revision of basic curricula have emerged. Moreover, modern technology, particularly robotic interventions and modern ICT can be integrated into patient care and nurses’ education. To face future challenges, the establishment of new care models, training programmes, nursing specialities and favourable policies related to COVID-19 care is crucial in the Sri Lankan context.

## Supporting information

S1 Checklist COREQ checklist in S1 Appendix. (DOC)
