# Supplementary material for: Nurses’ perspectives of taking care of patients with Coronavirus disease 2019: A phenomenological study
Source: PLoS One. 2021 Sep 3;16(9):e0257064. doi: 10.1371/journal.pone.0257064 (PMC8415609; doi:10.1371/journal.pone.0257064)
Supplement: S1 Checklist — (DOC) [file pone.0257064.s001.doc]

**S1 Appendix**

**Consolidated criteria for reporting qualitative studies (COREQ): 32-item checklist**

Developed from:

Tong A, Sainsbury P, Craig J. Consolidated criteria for reporting qualitative research (COREQ): a 32-item checklist for interviews and focus groups. *International Journal for Quality in Health Care*. 2007. 19 (6): pp. 349 – 357

| **No. Item** | **Guide questions/description** | **Reported on Page #** |
| --- | --- | --- |
| **Domain 1: Research team and reﬂexivity** |  |  |
| *Personal Characteristics* |  |  |
| 1. Interviewer/facilitator | Which author/s conducted the interview or focus group? | Page 4  Manuscript |
| 2. Credentials | What were the researcher’s credentials? E.g. PhD, MD | Title page |
| 3. Occupation | What was their occupation at the time of the study? | Title page |
| 4. Gender | Was the researcher male or female? | Title page |
| 5. Experience and training | What experience or training did the researcher have? | Title page |
| *Relationship with participants* |  |  |
| 6. Relationship established | Was a relationship established prior to study commencement? | Page 4  Manuscript  . |
| 7. Participant knowledge of the interviewer | What did the participants know about the researcher? e.g. personal goals, reasons for doing the research | Page 4  Manuscript |
| 8. Interviewer characteristics | What characteristics were reported about the interviewer/facilitator? e.g. Bias, assumptions, reasons and interests in the research topic | Page 4  Manuscript |
| **Domain 2: study design** |  |  |
| *Theoretical framework* |  |  |
| 9. Methodological orientation and Theory | What methodological orientation was stated to underpin the study? e.g. grounded theory, discourse analysis, ethnography, phenomenology, content analysis | Title page  Page 2 (abstract) & 4 (main document) |
| *Participant selection* |  |  |
| 10. Sampling | How were participants selected? e.g. purposive, convenience, consecutive, snowball | Page 2 & 4  Manuscript |
| 11. Method of approach | How were participants approached? e.g. face-to-face, telephone, mail, email | Page 2, 4 & 5  Manuscript |
| 12. Sample size | How many participants were in the study? | Page 2, 5 & 7  Manuscript |
| 13. Non-participation | How many people refused to participate or dropped out? Reasons? | Page 4  Manuscript .  (A purposive sample of nurses was initially recruited through social media (i.e. Facebook). Then, the snowballing sampling method was applied to recruit potential participants). Therefore, nurses only who expressed their willingness to participate in this study were included. Therefore, no non-participation |
| *Setting* |  |  |
| 14. Setting of data collection | Where was the data collected? e.g. home, clinic, workplace | Page 4  Manuscript  . |
| 15. Presence of non-participants | Was anyone else present besides the participants and researchers? | Page 4  Manuscript  Only the first researcher and interviewee were participated.  Inferred as “In-depth telephone interviews were conducted by the first researcher during June 2020, using an interview guide developed by the research team based on the literature and aim of this study. The telephone method helped to collect data during the curfew period with travel restriction. After identifying the potential participants, we distributed written information sheets and consent forms via electronic media (i.e., email and WhatsApp). Possible time for both parties was set.”. |
| 16. Description of sample | What are the important characteristics of the sample? e.g. demographic data, date | Page 2, 6, & 7, including Table 2  Manuscript |
| *Data collection* |  |  |
| 17. Interview guide | Were questions, prompts, guides provided by the authors? Was it pilot tested? | Page 4 & 5, including table 1.  Manuscript |
| 18. Repeat interviews | Were repeat interviews carried out? If yes, how many? | No |
| 19. Audio/visual recording | Did the research use audio or visual recording to collect the data? | Page 4 & 5  Manuscript |
| 20. Field notes | Were ﬁeld notes made during and/or after the interview or focus group? | Reflective notes were taken following the interviews |
| 21. Duration | What was the duration of the interviews or focus group? | Page 5  Manuscript |
| 22. Data saturation | Was data saturation discussed? | Page 4  Manuscript |
| 23. Transcripts returned | Were transcripts returned to participants for comment and/or correction? | Page 5  Manuscript |
| **Domain 3: analysis and ﬁndings** |  |  |
| *Data analysis* |  |  |
| 24. Number of data coders | How many data coders coded the data? | Page 6  Manuscript |
| 25. Description of the coding tree | Did authors provide a description of the coding tree? | Themes and sub-themes were provided in a table.  Table 3, Page 7 & 8. Manuscript |
| 26. Derivation of themes | Were themes identiﬁed in advance or derived from the data? | Derived from data.  Page 5 & 6  Manuscript |
| 27. Software | What software, if applicable, was used to manage the data? | Data were manually manipulated using a word processor. |
| 28. Participant checking | Did participants provide feedback on the ﬁndings? | Page 6  Manuscript |
| *Reporting* |  |  |
| 29. Quotations presented | Were participant quotations presented to illustrate the themes/ﬁndings? Was each quotation identiﬁed? e.g. participant number | Page 8 to 21  Manuscript |
| 30. Data and ﬁndings consistent | Was there consistency between the data presented and the ﬁndings? | Yes, there was.  Pages 8 to 21  Manuscript |
| 31. Clarity of major themes | Were major themes clearly presented in the ﬁndings? | Yes, they were.  From page 8-21 & Table 3 in pages 7 & 8.  Manuscript |
| 32. Clarity of minor themes | Is there a description of diverse cases or discussion of minor themes? | Discussion of major and minor themes  From page 22-28 and Table 3 in pages 7 & 8.  Manuscript |
